# Dynamic Defense against Stealth Malware Propagation in Cyber-Physical Systems: A Game-Theoretical Framework †

**DOI:** 10.3390/e22080894

**Published:** 2020-08-15

**Authors:** Kaiming Xiao, Cheng Zhu, Junjie Xie, Yun Zhou, Xianqiang Zhu, Weiming Zhang

**Affiliations:** Science and Technology on Information Systems Engineering Laboratory, National University of Defense Technology, Changsha 410073, China; zhucheng@nudt.edu.cn (C.Z.); xiejunjie06@gmail.com (J.X.); zhouyun@nudt.edu.cn (Y.Z.); xianqiangzhu@163.com (X.Z.); wmzhang@nudt.edu.cn (W.Z.)

**Keywords:** cyber-physical systems, stealth malware propagation, Stackelberg game, network interdiction, dynamic defense

## Abstract

Stealth malware is a representative tool of advanced persistent threat (APT) attacks, which poses an increased threat to cyber-physical systems (CPS) today. Due to the use of stealthy and evasive techniques, stealth malwares usually render conventional heavy-weight countermeasures inapplicable. Light-weight countermeasures, on the other hand, can help retard the spread of stealth malwares, but the ensuing side effects might violate the primary safety requirement of CPS. Hence, defenders need to find a balance between the gain and loss of deploying light-weight countermeasures, which normally is a challenging task. To address this challenge, we model the persistent anti-malware process as a shortest-path tree interdiction (SPTI) Stackelberg game with both static version (SSPTI) and multi-stage dynamic version (DSPTI), and safety requirements of CPS are introduced as constraints in the defender’s decision model. The attacker aims to stealthily penetrate the CPS at the lowest cost (e.g., time, effort) by selecting optimal network links to spread, while the defender aims to retard the malware epidemic as much as possible. Both games are modeled as bi-level integer programs and proved to be NP-hard. We then develop a Benders decomposition algorithm to achieve the Stackelberg equilibrium of SSPTI, and design a Model Predictive Control strategy to solve DSPTI approximately by sequentially solving an 1+δ approximation of SSPTI. Extensive experiments have been conducted by comparing proposed algorithms and strategies with existing ones on both static and dynamic performance metrics. The evaluation results demonstrate the efficiency of proposed algorithms and strategies on both simulated and real-case-based CPS networks. Furthermore, the proposed dynamic defense framework shows its advantage of achieving a balance between fail-secure ability and fail-safe ability while retarding the stealth malware propagation in CPS.

## 1. Introduction

Cyber-physical systems (CPS) are integrations of computation, networking, and physical processes, which have tremendously stimulated the development of contemporary critical infrastructures in different domains, such as energy, health care, communication, transportation, and manufacturing [1,2]. However, these integrations have unintentionally entailed new vulnerabilities, threats and challenges in safety and security [3,4]. The growing count of security-related incidents has drawn the increased focus on securing CPS.

Since advanced persistent threat (APT) attacks are usually well-planned and highly-customized, they have become a major and challenging threat for CPS defenders [5]. Specifically, the attackers carry out a long-term campaign with repeated attempts under the support of a highly organized and well-resourced malicious group who provides stealthy and evasive techniques; hence they are capable to adopt a series of advanced actions stealthily, persistently and detrimentally aiming at specific targets in critical infrastructures [5,6,7]. The computer worm Stuxnet, which was the first discovered malware to intelligently damage a nuclear power station, is the epitome of APT attacks [8], as well as other ensuing reported APT malware specimens, i.e., Duqu, Flame, Gauss [9], etc. Owing to the use of stealthy and evasive techniques (e.g., zero-day exploits, obfuscation techniques), these stealth malwares play a vital role in the attack process.

However, the existing security solutions for CPS usually make some of the following flawed assumptions which hinder themselves [10,11]: (i) the security problem is modeled as an one-shot attack-and-defense scenario, violating the feature of persistent actions in the stealth malware propagation; (ii) attackers only possess the ability of using a limited set of fixable exploits for relatively isolated attacks, which is far from the real practice for the stealth malware propagation where different types of stealthy and evasive techniques are used by attackers; and (iii) defenders’ response is confined to cleaning the detected threats and recovering the compromised nodes, neglecting the fact that unknown threats might be undetected, propagated, injected, and updated persistently by APT attackers to pursue long-term gains. Thus, conventional countermeasures, such as intrusion detection systems, enterprise-wide patching, and anti-virus techniques, usually fail to mitigate the risk and damage posed by stealth malwares.

More specifically, there is always a time-span of *unprotected exposure* when the defenders only adopt conventional countermeasures. Here, the unprotected exposure, which is accompanied by the attack-and-defense process, refers to the time-span between the detection of a certain intrusion and the complete deployment of a specified heavy-weight countermeasure (e.g., exploits patching, specialized anti-malware program). For defenders, their reaction duration towards newly detected threats directly determines the length of unprotected exposure. On the other hand, this unprotected exposure is crucial for the propagation of stealth malwares, since most valuable actions (e.g., updating, packing, targeting, propagating [12,13]) can be smoothly implemented in these time-spans. Given the fragility of CPS [14], attackers’ exploitation of unprotected exposure can bring them profitable returns and thus pose irreversible damages to the system. Unfortunately, the unprotected exposure is almost inevitable and it is difficult to reduce the time-span to a tolerable scale in complex CPS. The reason lies on the fact that a diversity of tools (e.g., zero-day exploits, public exploits) and techniques (e.g., polymorphism, metamorphism) are utilized in persistent actions by attackers, while defenders have to confront the complexity and bear the time-consuming nature of detecting threats accurately, as well as developing and deploying specified heavy-weight treatment countermeasures properly. As a consequence, traditional security approaches which ignore the unprotected exposure provide readily exploitable opportunities for APT attackers to propagate malwares persistently and then achieve malicious goals.

Light-weight countermeasures, on the other hand, can help retard the spread of malwares during the time-span of unprotected exposure, thereby mitigating overall damages and offering additional time for the development and deployment of heavy-weight treatment countermeasures [15,16]. Unfortunately, light-weight countermeasures are likely to incur safety risk because they are usually cursory and sometimes inaccurate; hence, the ensuing side effects are still inherent shortcomings of these countermeasures, especially for the CPS with both safety and security requirements. Safety and security which share identical goals of protecting systems from failures are two key properties of CPS [17]. Safety is protection against unintentional accidents, while security focuses on protecting systems from deliberate cyber attacks. For instance, containment techniques, such as firewalls, content filters, and routing blacklists [18,19], can be implemented to obstruct the propagation of worms whereas some legitimate communication which is necessary for systems’ normal operation might be blocked, as well. That is, when we try to maintain the security of a system using light-weight countermeasures, the safety requirements may be violated. This side effect is usually bearable in traditional information systems; however, the blockage of a critical legitimate control signal which is a safety requirement may lead to a catastrophic cascading in CPS [20]. However, very little research has studied the optimal allocating and scheduling strategy of light-weight countermeasures taking the safety requirement into consideration.

Since security and safety are interdependent with the requirements of one having effects on the other, the satisfaction of fail-secure and fail-safe ability requires a collaborative approach [20]. Here, *fail-secure* means that access or data will not fall into the wrong or malicious hands in a security failure, while *fail-safe* indicates that devices in a system will not endanger human lives or property when they fail. Hence, there is an urgent need for defenders to find an optimal trade-off between the gain and loss of deploying light-weight countermeasures in CPS, thereby achieving a balance between fail-secure and fail-safe ability. To address this problem, we model the persistent attack-and-defense process between the CPS defender and the attacker as a dynamic Stackelberg game. The attacker aims to stealthily penetrate the CPS at the lowest cost (e.g., time, effort) by selecting optimal network links to spread. On the other hand, the defender who has limited capabilities to detect stealth malwares aims to maximize the fail-secure ability, i.e., retarding the malware epidemic as much as possible by optimally allocating light-weight countermeasures until the development and deployment of heavy-weight countermeasures. Meanwhile, safety requirements of CPS are considered in the form of constraints in the defender’s decision model, which reflects the primary importance of fail-safe ability. The main contributions of this paper are:

1) We propose the static shortest-path tree interdiction (SSPTI) game, model it as a bi-level integer program (BLIP), and prove its NP-hardness. A Benders decomposition algorithm (S-BD) is then developed to achieve its Stackelberg equilibrium.
2) We extend the SSPTI to a multi-stage dynamic shortest-path tree interdiction (DSPTI) game to support the of real-time decision-making in the persistent attack-and-defense process, and design a *model predictive control* (MPC) strategy for the defender. An 1+δ approximation algorithm is proposed for the defender to achieve local optimality, thereby expanding the solvable scale of the problem.
3) The evaluation results demonstrate that the proposed approximation algorithm can enlarge the solvable scale of problems with an order of magnitude improvement (reporting an increase from less than 100 nodes to more than 3000 nodes) and reduce the resources consumption by 60%.
4) The performance of proposed MPC strategy is better than existing strategies on both simulated and real-case-based CPS networks. A lower steady infection rate and a higher ratio of giant component can be achieved by MPC strategy simultaneously, which means it can help retard the spread of malwares and the cascade of devices failure at the same time.

The reminder of this paper is organized as follows. Section 2 provides the related work, and Section 3 introduces the network model of CPS and the Stackelberg game model between the defender and the attacker aiming to penetrate the CPS. In Section 4, SSPTI game is formulated as a BLIP, and an exact algorithm is designed for it. The game is then extended to a dynamic version in Section 5, and an MPC strategy is designed for DSPTI. Section 6 reveals the performance of proposed algorithms and strategies. Section 7 concludes this paper.

## 2. Related Works

### 2.1. Security Countermeasures

To address the challenge of CPS security, researchers have paid a great attention on the security issues of malwares propagation, such as propagation modeling, prevention, detection, and mitigation [20,21,22]. However, the unprotected exposure is usually neglected and few of them have considered the trade-off between safety and security when using light-weight countermeasures.

Heavy-weight countermeasures have been studied and applied in practice for decades, such as exploits patching [23], nodes or edges removing [24], and anti-malware program updating [20]. Many of those studies use control-theoretic mitigation strategies. Bloem et al. proposed an optimization problem of malware removal or path deployment and they captured the trade-off between the infection speed and patching costs by using an optimal control framework [25]. In Reference [26], a quarantine control method was developed so as to study the propagation and inhibition of virus in time-varying networks. In order to suppress SIS epidemics in networks, Scaman et al. [24] designed a series of control-theoretic resource allocation strategies. In Reference [23], a general framework was developed to achieve optimal patching policies against malware epidemics, in which the issue of disseminating security patches in a large resource-constrained heterogeneous mobile network was taken into account.

On the other hand, there are some researchers that committed to use light-weight countermeasures to secure network systems. For instance, Yau et al. [15] proposed a tolerance mechanism to minimize the damage caused by distributed denial of service (DDoS) attacks. The core of the mechanism is the max-min fair server centric throttling, and the minimization of DDoS damage leads to the loss of legitimate packet. In the defense of selective forwarding attack, by deploying the randomly selected single checkpoint node in the system, the forwarding misbehavior of the malicious vertex can be detected which was a kind of light-weight countermeasure [27]. In order to mitigate TCP SYN flooding attacks, Mohammadi et al. [28] developed an SDN-based light-weight countermeasure in the controller level, in which ongoing TCP connection requests will be checked and malicious hosts will be blocked. In Reference [29], a DoS attacks defense framework for SDN/OpenFlow networks was developed in a protocol-independent manner, where attack traffic is identified by a packet filter.

### 2.2. Network Interdiction

Network interdiction problems are usually modeled as games involve two players who commonly have opposite utility functions, which have been wildly applied in traditional security problems, such as nuclear smuggling interdiction [30], terrorist attack defense [31], facilities fortification [32], and illegal products detection [33]. Shortest path network interdiction is a typical interdiction game, which was introduced in 1977 by Fulkerson and Harding [34]. In this problem, the interdictor wishes to maximize the shortest path the evader can achieve using limited resources, and the length of arc was assumed to increase linearly with the amount of resource allocated. In the model of mixed integer version proposed by Wood [35], the decision variable became binary and Benders decomposition method was proposed to solve it. After that, variants of network interdiction games with new features were developed. For instance, Bayrak and Bailey proposed a shortest path network interdiction game with information asymmetry, in which the interdictor and the evader have different levels of network information [36]. In Reference [37], the vulnerability of multiple-commodity system to multiple disruptions was studied, where the formulation of finding defense strategies at minimal cost that maintain a high level of demand satisfaction across all commodities was proposed. Borrero et al. investigated sequential interdiction problem in which the interdictor has incomplete initial information about the network, including its structure and arc costs [38]. In their work, learning method was used so that the interdictor can learn about the network structure and arc costs by observing the evader’s actions. A dynamic shortest path network interdiction without goal information was proposed in Reference [39], where goal recognition methodology was introduced to help the inerdictor make a flexible resource assignment.

Recently, some researchers have payed more attention to the development of security strategies of information systems or computer networks within the framework of game theory. In Reference [40], a resilient control problem in CPS was modeled as a two-level receding-horizon dynamic Stackelberg game between the system operator and human adversaries. Panaousis et al. [41] proposed a decision support approach for cyber-security issues in the framework of game theory, in which they modeled non-cooperative cyber-security control games between the network defender and the attacker who can exploit different vulnerabilities in the computer network. An attack graph interdiction game was developed by Nandi et al. in [42] aiming to protect organizations from cyber attacks. In this game, the defender aims to find an optimal affordable subset of links to deploy countermeasures, while the attacker aims to penetrate the network through the feasible path in attack graph. In order to take attackers reaction into account, Durkota et al. [43] studied the optimal strategy making of placement of honeypots in a network using a game-theoretic approach. Using the Bayesian Stackelberg Game, Zeng, et al. [22] studied the problem of infrastructure network protection under asymmetry information in which multiple attackers types were considered and Bayesian Stackelberg game was introduced to model this problem.

## 3. Network Model and Stackelberg Game

In this section, we introduce the network model of CPS and the Stackelberg game model under the threat of stealth malware epidemic. Basic assumptions about CPS and settings of the game are presented. Table 1 presents the notations of main parameters and variables in this paper.

### 3.1. Network Model

**Figure 1:**
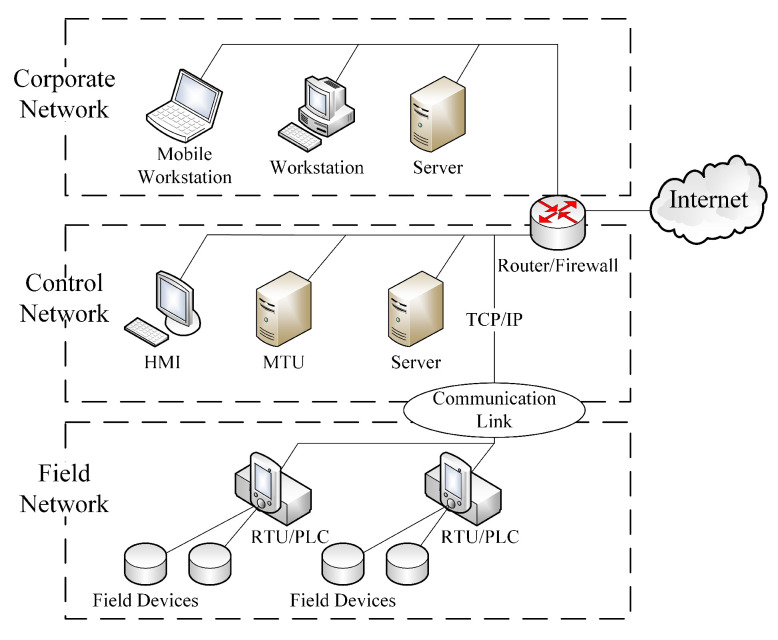
A typical CPS network with three layers.

A CPS network usually includes three layers, i.e., a corporate network, a control network and a field network [44]. As shown in Figure 1, in field network, field devices are instrumented by means of sensors and actuators. The remote terminal unit (RTU) provides a communication interface for field devices, and its role is played by a programmable logic controller (PLC) in many scenarios. The communication link connects the control and filed networks based on various communication techniques, e.g., wire, fiber optic, radio, microwave [45]. The control network contains several servers with various purposes, such as the master terminal unit (MTU) for data collection and periodically RTU polling. Operators access recorded data, check reported alarms and issue commands via a human-machine interfaces (HMI) in this layer. The corporate network usually includes workstations for engineers and system administrators which are generally connected to the Internet and sometimes to the control network via a router.

**Figure 2:**
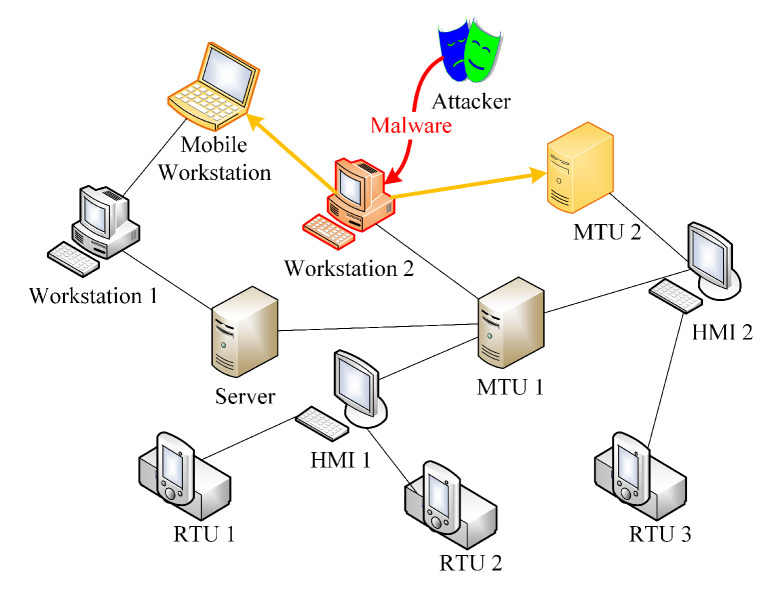
An example of malware propagation in cyber-physical systems (CPS).

Although the CPS network is full-connected physically, strict access control and designed communication patterns let the actual network structure not fully connected under operating conditions. Figure 2 gives an abridged view on the topology of an operating CPS network, where links present the permitted or designed communication patterns between CPS devices rather than all possible connections. It is worth noting that any traffic violating the those communication patterns will be treated as abnormal or illegitimate in CPS [45], which is a significant difference between CPS and traditional information systems.

In general, we present the topology of CPS as an undirected graph G=(V,E), where *V* is the set of CPS devices and *E* is the set of links connecting devices in *V*. Each link e∈E has a weight $w_{e}\geq 0$ which presents its significance to CPS safety (vector form w). The cost for an attacker to propagate a malware through the link *e* is denoted by $c_{e}\geq 0$ (vector form c). The allocation of light-weight countermeasures on a link *e* is assumed to make a delay of $d_{e}>0$ (vector form d) to the malware propagation on this link, while resulting a loss of $w_{e}$ in CPS safety. These parameters can be determined by security and safety maintenance personnel of CPS.

### 3.2. Stackelberg Game Model

In a typical defense scenario against stealth malware propagation in CPS, the attacker initially selects a set of devices to infect, while the CPS defender usually operates a malware detection system providing with an incomplete infection distribution. When the information of newly-detected threats is reported, the defender must implement countermeasures accordingly; however, heavy-weight countermeasures cannot be implemented immediately due to the existence of unprotected exposure. Then, the issue of how to use light-weight countermeasures to retard the epidemic tends to be crucial to CPS defenders. It is clear that containment techniques can block illegal communication thereby stopping the spread of malwares through certain links, but may put the system at risk due to the blockage of some crucial control signals for safety concerns. In practice, CPS defenders can be classified according to their preference; that is, the one who gives the priority to security is called *fail-secure defender*, and a *fail-safe defender* refers to the one who has preference for safety.

To exemplify this, an instance of the defense scenario is given in Figure 2, where workstation 2 has been infected with a kind of malware from an attacker and two copies of this malware has been stealthily spread to mobile workstation and MTU 2. The defender is supposed to know the abnormal condition of workstation 2 based on its detection system, but more time is needed to analyze the malware sample, develop exploits patching, and deploy anti-malware programs. Hence, the defender should firstly implement light-weight countermeasures. A fail-safe defender is likely to isolate the detected infectious device, though its neighbors (i.e., mobile workstation, MTU 1, and MTU 2) may have been infected, as well. That is, no light-weight countermeasures will be taken on those neighbors such that all control signals deriving from them can be transmitted to the remained network, i.e., fail-safe ability can be maintained as much as possible. Apparently, the undetected copies of this malware on mobile workstation and MTU 1 will pose a long-term threat to the security of this CPS. On the other hand, if the defender gives the priority to security, the initially infected workstation 2 and its neighbors who have the risk of infection should be isolated at the same time so as to avoid further infections deriving from possible infected neighbors. In this way, the defender tries its best to prevent illegal access and data leakage; however, the remained network becomes too fragmented to function normally and the blockage of certain control signals (e.g., emergency cut-off signal, load balancing signal, etc.) may increase the risk of safety. Therefore, it is urgent for the defender to achieve a balance between fail-secure and fail-safe ability when adopting light-weight countermeasures to retard the malware propagation.

To this end, we consider a Stackelberg game in the CPS under the threat of stealth malware propagation, named *Shortest-path Tree Interdiction Game*. There are two players in this game, i.e., a defender and an attacker. That is,

(1) $$N_{p}=\left\{\mathrm{Defender},\mathrm{Attacker}\right\}.$$

The defender takes action first to allocate defense resources to a set of links, and then the attacker starts to implement its malicious propagation plan based on the observation of defender’s action. The strategy sets of the defender and attacker are defined as $S_{D}$ and $S_{A}$ as follows:

(2) $$S_{D}=\left\{s_{D}:=x|x\in X\right\},$$

(3) $$S_{A}=\left\{s_{A}:=y|y\in Y\right\},$$

which denotes by *X* the feasible set of x for the defender, and we let *Y* be the feasible set of y for the attacker. The defender acts as the leader in this game and can observe a distribution of malware instances in the system based on the detected results, though this observation is usually incomplete. The attacker, on the other hand, acts as a follower and is assumed to know the actions of the defender. Here, the follower knows the whole information of its opponent, and this kind of worst-case analysis is proper for defenders who need robust defense strategies.

#### 3.2.1. Utility Function for Attacker and Defender

The target of the attacker is to stealthily penetrate the CPS at the lowest cost (e.g., time, effort) by selecting optimal network links to spread. According to the nature of tree-structure spreading pattern, the propagation problem is modeled as a shortest-path tree problem with a root vertex $v_{s}$, i.e., the initial infectious device. That is, the aim of the attacker is to minimize the sum of the path lengths (i.e., penetrating time) from $v_{s}$ to each vertex $v_{i}\in V$. For multiple initially infected devices, we can introduce an equivalent root vertex and then connect the initial infectious devices to it by links given $c_{e}=0,\;w_{e}=+\infty$. By contrast, the defender aims to optimally allocate light-weight countermeasures to several links thereby maximizing the attacker’s total penetrating time. In this game, the utility function of the defender is just the opposite of the attacker’s payoff and they are both linear; hence, this is a typical two-player zero-sum game.

#### 3.2.2. Functional Assurance Constraints

To ensure the safety of CPS, some functional assurance constraints are introduced during the defender’s decision-making process of pursuing fail-secure ability. Since allocation of light-weight countermeasures can cause the loss of safety weight on links, the defender can make an upper bound of total loss *R* on weight such that the defender can assure the whole system’s safety according to the weight vector w and safety requirements. In some cases, the safety of CPS includes the special safety needs of some certain components, such as a data server or workstation with higher safety needs level. As to these cases, each critical component can be similarly assigned an individual upper bound of weight loss $r_{i}$ relating to vertex $v_{i}$.

#### 3.2.3. Static and Dynamic Version of the Game

A typical Stackelberg game is usually static, i.e., both players adopt a once-and-for-all decision at the beginning of the game. In the SPTI game, if the attacker makes an entire spread plan and does not change it during the spreading process, the interdiction action of the defender can be designed as a static one. This game is defined as static SPTI game, and the equilibrium result provides the defender with a static allocation decision which is optimal when the attacker does not change its spread plan. Furthermore, when the defender face a cunning attacker who takes adaptive actions according to its observation of the countermeasure implementation, dynamic defending in an observe-and-response manner becomes indispensable to a defender dedicating to an efficient defense. This dynamic SPTI game is a kind of extensive game, which provides defenders with a more practical and operational approach to optimal interdiction compared with the static one.

## 4. Static Shortest-Path Tree Interdiction Game

We first study the static version of the SPTI game, where both players take once-and-for-all strategies during the game. That is, the defender observes the distribution of malware and allocates defense resources all at once, while the attacker implements its designed propagation plan consistently. Hence, this static game is a typical two-player zero-sum Stackelberg game.

### 4.1. Bi-Level Integer Program Formulation

In order to obtain the equilibrium of players, we first need to formulate the game mathematically. Since the sets of strategies of players deriving from the constraints in the decision models are implicit in the proposed Stackelberg game, it is usually formulated as a BLIP so as to achieve the Stackelberg equilibrium [35]. Let $x_{e}$ be the decision variable of the defender (vector form x), where $x_{e}=1$ if the link *e* is interdicted by the defender using light-weight countermeasures; otherwise $x_{e}=0$. Similarly, the attacker’s decision variable $y_{e}=1$ if the link *e* is chosen by the attacker as an edge in the propagation tree of malwares; otherwise $y_{e}=0$; hence, each y denotes a spanning tree from $v_{s}$. $NS(v_{i})$ denotes the set of links connected to vertex $v_{i}$. Let U(x,y) denote the utility function of this game under the network setting (i.e., c and d). Let *Y* denote the set of all y vectors, i.e., the set of all spanning trees from $v_{s}$ in *G*. Hence, we can formulate the game as follows:

$$\begin{array}{c} \left[\mathrm{SSPTI}\right]\;u^{*}=\max_{x}\min_{y}\;U\left(x,y\right) \end{array}$$

(4) $$\begin{array}{c} s.t.\;\;y\in Y, \end{array}$$

(5) $$\begin{array}{c} \sum_{e\in E}w_{e}x_{e}\leq R, \end{array}$$

(6) $$\begin{array}{c} \sum_{e\in NS(i)}w_{e}x_{e}\leq r_{i}\;\forall \;v_{i}\in V, \end{array}$$

where $x,y\in {\left\{0,1\right\}}^{\left|E\right|}$, $u^{*}$ is the Stackelberg equilibrium of this game. Constraint (4) shows that the propagation of malwares should follow a tree-structure pattern. Constraints (5) and (6) are functional assurance constraints, which mean that the allocation of light-weight countermeasures on the whole CPS and each CPS device should not exceed the upper bound *R* and $r_{i}$, respectively.

SSPTI is a BLIP, where the inner-level problem of the attacker is a shortest-path tree problem and the outer-level problem of the defender introduces knapsack Constraints (5) and (6), which adds to its complexity. Without surprise, SSPTI is a NP-hard problem, and the NP-hardness of it is analyzed in Theorem 1.

**Theorem** **1.** *The SSPTI problem is NP-hard.*

**Proof** **of** **Theorem** **1.** Firstly, we introduce a problem of finding a subset *K* of links such that $\sum_{e\in K}w_{e}\leq R$ and whose removal from *G* results in the largest increase in the length of the shortest-path tree from root $v_{s}$ to each vertex, called k-most-vital-links problem (KMVP). It is clear that KMVP is a special case of SSPTI problem. Note that we omit the Constraint (6) in the proof because that SSPTI problem without Constraint (6) is equivalent to setting $r_{i}\geq R,\;\forall i\in V$; thus is a special case of SSPTI problem.

We then prove that KMVP is NP-hard by showing that a k-most-vital-links recognition problem (KRP) related to it is NP-complete. The KRP is:

INPUT: A undirected graph G=(V,E); w≥0, c≥0; R≥0; $v_{s}\in V$.
OUTPUT: Yes, if there exists a set K⊆E such that $\sum_{e\in K}w_{e}\leq R$ and the total length of the shortest-path tree from $v_{s}$ in G(V,E\K) is ≥U; no, otherwise.

The existence of a polynomial algorithm for KMVP would imply the existence of a polynomial algorithm for KRP. Hence, we then demonstrate that KRP is NP-complete by reducing the following knapsack problem (KNAP) to it. The KNAP as follows is known to be NP-complete [46].

INPUT: $a_{j}$, $b_{j}\geq 0$ for j=1,2,⋯,n; A,B≥0.
OUTPUT: Yes, if there exists a set S⊆(1,2,⋯,n) such that $\sum_{j\in S}a_{j}\geq A$ and $\sum_{j\in S}b_{j}\leq B$; no, otherwise.

**Figure 3:**
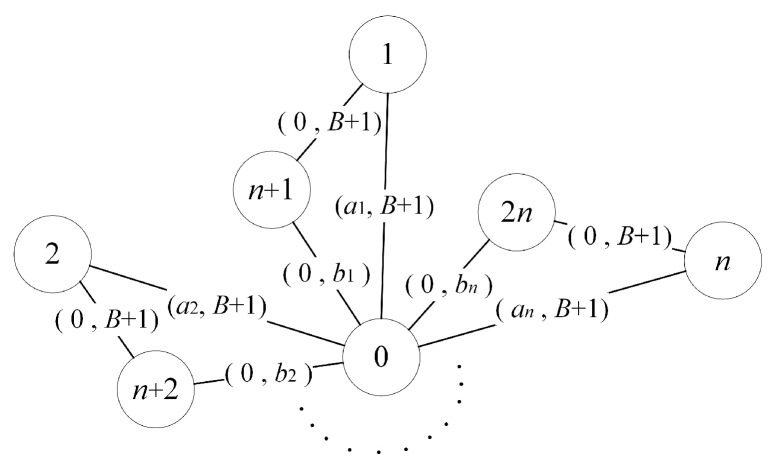
A portion of the constructed graph in the reduction process.

The reduction process is as follows. We construct an undirected graph with 2n+1 vertexes labeled as 0,1,2,⋯,2n. As shown in Figure 3, there is a link between vertex 0 and *j* (for j=1,2,⋯,n) with length $a_{j}$ and removal cost B+1. Vertex n+j (for j=1,2,⋯,n) is only adjacent to vertexes 0 and *j*, where the lengths of link (0,n+j) and (j,n+j) are both 0, and the removal costs are $b_{j}$ and B+1, respectively.

Finally, we consider the problem KRP defined on this graph with R=B, U=A, and $v_{s}=0$. It is observed that none of links (0,j) and (j,n+j) for j=1,2,⋯,n can be members of *K*. Therefore, we may assume that only links (0,n+j) for j=1,2,⋯,n are removed. For convenience, link (0,n+j) is simply denoted by index *j*, and let *K* be any set of removed links that does not violate the budget constraint $\sum_{e\in K}w_{e}\leq R$. Then, $\sum_{j\in K}b_{j}\leq B$, and the total length of the shortest-path tree from vertex 0 becomes $\sum_{j\in K}a_{j}$. That is, there is a one to one correspondence between solutions of KRP and solutions of KNAP. □

### 4.2. Algorithm Design of SSPTI

Solving the BLIP is commonly recognized as a difficult task, and has attracted many efforts from researchers [47]. The common base of existing resolution methods for BLIP is the single-level reformulation process (including linear dual approach, Benders decomposition method, etc.), which transforms bi-level programming to the single-level one. Since SSPTI is a BLIP which cannot be solved directly without proper reformulation, we propose a decomposition algorithm for it by applying Benders decomposition approach [35] and then prove its correctness. Before introducing the algorithm S-BD, some additional denotations are given as follows.

Let $\hat{y}\in {\left\{0,1\right\}}^{\left|E\right|}$ denote the incidence vector corresponding to a certain spanning tree from root $v_{s}$ in *G*. Let $\hat{Y}$ denote a collection of $\hat{y}$. For simplicity, we refer to $\hat{y}$ as a spanning tree and to $\hat{Y}$ as a set of spanning trees. For a certain spanning tree $\hat{y}$, we introduce a hierarchy vector h to reflect the weight of each link when accumulating the length of path from $v_{s}$ to each vertex. The hierarchy of link e=(i,j) is defined as the number of descendants of vertex *i*, where *i* is defined as the parent of *j* without loss of generality. That is, if ${\hat{y}}_{e}=0$ then $h_{e}=0$, and if ${\hat{y}}_{e}=1$, the value of $h_{e}$ is the hierarchy of link *e*. Hence, for a certain spanning tree $\hat{y}$, we can explicitly express the utility function as:

(7) $$U\left(x,\hat{y}\right)=c^{T}H\hat{y}+x^{T}DH\hat{y},$$

where D=diag(d), and H=diag(h).

Based on this explicit expression of utility function, we can reformulate program [SSPTI] as the following decomposed Master-Sub programs:

$$\begin{array}{c} \left[\mathrm{Master}\left(\hat{Y}\right)\right]\;z_{\hat{Y}}=\max_{x}\;z \end{array}$$

(8) $$\begin{array}{c} s.t.\;\;\;z\leq c^{T}H\hat{y}+x^{T}DH\hat{y}\;\forall \;\hat{y}\in \hat{Y}, \end{array}$$

(9) $$\begin{array}{c} \sum_{e\in E}w_{e}x_{e}\leq R, \end{array}$$

(10) $$\begin{array}{c} \sum_{e\in NS(i)}w_{e}x_{e}\leq r_{i}\;\forall \;v_{i}\in V,\\ \left[\mathrm{Sub}\left(\hat{x}\right)\right]\;\;z_{\hat{x}}=\min_{y}\;U\left(\hat{x},y\right) \end{array}$$

(11) $$\begin{array}{c} s.t.\;\;\;\;y\in Y. \end{array}$$

For fixed $x=\hat{x}$, there is always a solution $\hat{y}\in Y$ to [Sub($\hat{x}$)], which is equivalent to a solution to the inner minimization problem of [SSPTI]. Besides, [Sub($\hat{x}$)] is a shortest-path tree problem which can be solved in polynomial time [48]. Problem [Master($\hat{Y}$)], on the other hand, is an equivalent reformulation of [SSPTI] when $\hat{Y}=Y$. Therefore, if one could enumerate all shortest-path spanning trees $\hat{y}\in Y$ under each $\hat{x}$ by solving [Sub($\hat{x}$)], the optimal solution of [SSPTI] could be obtained by solving [Master($\hat{Y}$)].

The direct idea of enumerating all shortest-path trees is still time-consuming; however, we can solve [SSPTI] optimally by sequentially generating only a small fraction of the trees in *Y* in a decomposition manner thereby increasing the efficiency. Given an allowable optimality gap ϵ, the S-BD algorithm is described in Algorithm 1. After that, Theorem 2 demonstrates the correctness of S-BD algorithm.
**Algorithm 1** S-BD: Benders Decompostion for SSPTI1:   Initialize $\hat{Y}\leftarrow \emptyset$, $\underline{z}\leftarrow -\infty$, $\bar{z}\leftarrow +\infty$, $\hat{x}\leftarrow 0$, ϵ2:   **while** $\bar{z}-\underline{z}\leq \epsilon$ **do**3:         Solve [Sub($\hat{x}$)] for $\hat{y}$ and objective value $z_{\hat{x}}$4:         $\hat{Y}\leftarrow \hat{Y}\cup \hat{y}$5:         **if** $\underline{z}<z_{\hat{x}}$ **then** $\underline{z}\leftarrow z_{\hat{x}}$6:         Solve [Master($\hat{Y}$)] for $\hat{x}$ and objective value $z_{\hat{Y}}$7:         $\bar{z}\leftarrow z_{\hat{Y}}$8:   Return $x^{\epsilon }\leftarrow \hat{x}$

**Theorem** **2.** *Algorithm 1 correctly solves SSPTI.*

**Proof** **of** **Theorem** **2.** Firstly, let *X* be the feasible region of x in SSPTI. Since $\hat{Y}\subseteq Y$ the following is valid for any x∈X: $\min_{y\in \hat{Y}}U\left(x,y\right)\geq \min_{y\in Y}U\left(x,y\right)$. Hence, it is clear that $\max_{x\in X}\min_{y\in \hat{Y}}U\left(x,y\right)\geq \max_{x\in X}\min_{y\in Y}U\left(x,y\right)$ is valid, i.e., [Master($\hat{Y}$)] provides an upper bound to [SSPTI].

Secondly, $\max_{x\in X}\min_{y\in Y}U\left(x,y\right)\geq \min_{y\in Y}U\left(\hat{x},y\right)$ is apparently satisfied for any specific $\hat{x}$, which means the solution to [Sub($\hat{x}$)] is a lower bound of [SSPTI].

Therefore, if $\bar{z}=\underline{z}$ (i.e., ϵ=0) is valid with a solution $\hat{x}$, i.e., $\max_{x\in X}\min_{y\in \hat{Y}}U\left(x,y\right)=\min_{y\in Y}U\left(\hat{x},y\right)$, then we have

(12) $$\max_{x\in X}\min_{y\in \hat{Y}}U\left(x,y\right)=\max_{x\in X}\min_{y\in Y}U\left(x,y\right)=\min_{y\in Y}U\left(\hat{x},y\right).$$

That is, $\hat{x}$ is the optimal solution for the defender. □

## 5. Dynamic Shortest-Path Tree Interdiction Game

In the model setting of SSPTI game, both players adopt stationary strategies which sometimes violate the fact that a cunning attacker is likely to adjust its propagation plan according to the dynamic environment thereby achieving a better payoff. The defender, on the other hand, needs to take actions in an observe-and-react loop rather than take an one-shot defense because of the exist of unprotected exposure and forthcoming malicious events. The model of SSPTI obviously cannot meet those dynamic and adaptive needs of the interdictor. In order to meet the fact of real-time defense-and-attack decision-making, this section extends SSPTI to a multi-stage dynamic shortest-path tree interdiction (DSPTI) game, which can be modeled as an extensive-form game.

### 5.1. Extending SSPTI to the Dynamic Game

In contrast to the SSPTI game, both players can adopt an observe-and-response action rather than an once-and-for-all decision in real cyber-combat. That is, once newly infectious devices are detected, the defender will allocate certain light-weight countermeasures to certain links. Similarly, the attacker can redesign its propagating tree based on the observation of its opponent’s actions. Based on this practical realization, we propose the following DSPTI game.

The DSPTI game in extensive form is an ordered vector,

(13) $$\Gamma_{D}=\left(N_{p},V_{g},E_{g},s_{g},{\left(V_{gi}\right)}_{i\in N_{p}},O,u\right),$$

where $N_{p}$ is a finite set of players (i.e., defender and attacker). $\left(V_{g},E_{g},s_{g}\right)$ denotes a tree called the game tree from root $s_{g}$, which is determined by the initial infected vertex $v_{s}$, the graph *G* and the functional assurance Constraints (5) and (6). ${\left(V_{gi}\right)}_{i\in N_{p}}$ is a partition of the set of vertexes that are not leaves, denoting the decision vertex of player *i*, and in this game the attacker and the defender make decisions in an alternating manner. Let τ be the number of decisions that each player has to make during this game. *O* denotes the set of possible game outcomes and *u* is a function associating every leaf of the tree with a game outcome in the set *O*, i.e., the utility function that accumulates all path lengths from infected root $v_{s}$ to all each vertex for each game outcome. We can also demonstrate that the DSPTI game is NP-hard shown in Theorem 3.

**Theorem** **3.** *The DSPTI game is NP-hard.*

**Proof** **of** **Theorem** **3.** Firstly, we introduce a problem of finding a sequence of subsets $K_{t}$ (for t=1,2,⋯,τ) of links such that $\sum_{t=1}^{\tau }\sum_{e\in K_{t}}w_{e}\leq R$ and whose removal from *G* sequentially results in the largest increase in the length of the shortest-path tree from root $v_{s}$ to each vertex, called dynamic k-most-vital-links problem (DKMVP). It is clear that DKMVP is a special case of DSPTI problem.

We then prove that DKMVP is NP-hard by showing that a dynamic k-most-vital-links recognition problem (DKRP) related to it is NP-complete. The DKRP is:

INPUT: A undirected graph G=(V,E); w≥0, c≥0; R≥0; $v_{s}\in V$.
OUTPUT: Yes, if there exists a sequence of subsets $K_{t}\in E$ (for t=1,2,⋯,τ) such that $\sum_{t=1}^{\tau }\sum_{e\in K_{t}}w_{e}\leq R$ and the accumulated traversing length of the shortest-path tree from $v_{t}$ in $G(V,E\backslash \left\{K_{1},K_{2},\cdots ,K_{t}\right\})$ for t=1,2,⋯,τ is ≥U; no, otherwise.

Hence, we then demonstrate that DKRP is NP-complete by reducing the knapsack problem (KNAP) in Theorem 1 to it. The reduction process is as follows.

We reuse the graph shown in Figure 3. Similarly, we consider the problem DKRP defined on this graph with R=B, U=A, and $v_{s}=0$. It is observed that none of links (0,j) and (j,n+j) for j=1,2,⋯,n can be members of any $K_{t}$ for t=1,2,⋯,τ. Therefore, we may assume that only links (0,n+j) for j=1,2,⋯,n are removed. For convenience, link (0,n+j) is simply denoted by index *j*, and let $\left\{K_{1},K_{2},\cdots ,K_{\tau }\right\}$ be any sequence of sets of removed links in the time period from 1 to τ that does not violate the budget constraint $\sum_{t=1}^{\tau }\sum_{e\in K_{t}}w_{e}\leq R$. It is obvious that $K_{t}=\emptyset$, ∀t∈{2,3,⋯,τ}; hence, $\sum_{t=1}^{\tau }\sum_{e\in K_{t}}w_{e}=\sum_{j\in K_{1}}b_{j}\leq B$, and the total length of the shortest-path tree from vertex 0 becomes $\sum_{j\in K_{1}}a_{j}$. That is, there is a one to one correspondence between solutions of DKRP and solutions of KNAP. □

### 5.2. A Model Predictive Control Strategy for DSPTI

In addition to its NP-hardness, it is impossible to achieve the Stackelberg equilibrium for the defender before the end of the game due to the uncertainty from incomplete malware detection and the lack of future data. Traditional exponential-state backward recursion approaches to other kinds of multi-stage interdiction games, such as the dynamic-programming algorithm for dynamic shortest-path interdiction game [49], are not capable of tackling the real-time problem in which no future observation information could be obtained in advance and then used in the backward recursion procedure. To address this, drawing on ideas of modern control theory and methodology, we propose a MPC strategy for DSPTI game with respect to the requirement of real-time decision-making. The idea of MPC strategy derive from the advanced approach of process control, i.e., Model Predictive Control, which allows the current stage to be optimized while keeping the future stages in account [50]. The detection system of malwares serves as the system model in this MPC strategy, and the optimizer is defined to solve a local-greedy SSPTI problem (LG-SSPTI) in a rolling horizon manner.

Once newly infectious devices are detected, a new decision round *t* will be started by the defender and its opponent. According to the idea of MPC, we need to optimally solve the SSPTI at this round *t*, and only implements decisions on the neighbor links of $v_{s}$. However, this simple idea is wasteful in the use of computing resource since useless decisions on non-neighbor links of $v_{s}$ are made and SSPTI itself is NP-hard. Therefore, we introduce the local-greedy SSPTI problem first. The approximate decision of the defender at stage *t* is made by solving the following LG-SSPTI problem:

$$\begin{array}{c} \left[\mathrm{LG}-\mathrm{SSPTI}(t)\right]\;u^{*}\left(t\right)=\max_{x^{t}}\min_{y^{t}}\;U\left(x^{t},y^{t}\right) \end{array}$$

(14) $$\begin{array}{c} s.t.\;\;y^{t}\in Y, \end{array}$$

(15) $$\begin{array}{c} \sum_{e\in E}w_{e}x_{e}^{t}\leq R^{t}, \end{array}$$

(16) $$\begin{array}{c} \sum_{e\in NS(i)}w_{e}x_{e}^{t}\leq r_{i}^{t}\;\forall \;v_{i}\in V, \end{array}$$

(17) $$\begin{array}{c} x_{e}^{t}=0\;\forall \;e\notin NS(v_{s}), \end{array}$$

where $x^{t},y^{t}\in {\left\{0,1\right\}}^{\left|E\right|}$, $u^{*}\left(t\right)$ is the Stackelberg equilibrium of this game, and $NS(v_{s})$ is the set of links directly connected to $v_{s}$. Constraint (14) denotes that the propagation of malwares should follow a tree-structure pattern. Constraints (15) and (16) are functional assurance constraints at stage *t*, where $R^{t}$ and $r_{i}^{t}$ denotes the available total and individual resources left after stage t−1. Constraint (17) denotes a local-greedy allocation constraint that only links connected to vertex $v_{s}$ can be selected to allocate light-weight countermeasures at stage *t*.

**Theorem** **4.** *The LG-SSPTI problem is NP-hard.*

**Proof** **of** **Theorem** **4.** Different from the SSPTI problem, Constraint (17) introduces new features. Hence, we first introduce a problem of finding a subset *K* of links which connected to $v_{s}$ such that $\sum_{e\in K}w_{e}\leq R$ and whose removal from *G* results in the largest increase in the length of the shortest-path tree from root $v_{s}$ to each vertex, called local greedy k-most-vital-links problem (LG-KMVP). It is clear that LG-KMVP is a special case of LG-SSPTI problem. Due to the reason in the proof of Theorem 1, we omit the Constraint (16) in the proof.

We then prove that LG-KMVP is NP-hard by showing that a local greedy k-most-vital-links recognition problem (LG-KRP) related to it is NP-complete. The LG-KRP is:

INPUT: A undirected graph G=(V,E); w≥0, c≥0; R≥0; $v_{s}\in V$.
OUTPUT: Yes, if there exists a set $K\subseteq NS(v_{s})$ such that $\sum_{e\in K}w_{e}\leq R$ and the total length of the shortest-path tree from $v_{s}$ in G(V,E\K) is ≥U; no, otherwise.

The existence of a polynomial algorithm for LG-KMVP would imply the existence of a polynomial algorithm for LG-KRP. Hence, we then demonstrate that LG-KRP is NP-complete by reducing the above-mentioned NP-complete problem, i.e., knapsack problem (KNAP) in Theorem (1), to it.

The reduction process is as follows. Since links $\left(j,n+j\right)\notin NS\left(v_{s}\right)$ for j=1,2,⋯,n, they cannot belong to *K*. Thus, we can still reuse the graph shown in Figure 3 though the removal cost of links (j,n+j) for j=1,2,⋯,n can be set as any value Then, we consider the problem LG-KRP defined on this graph with R=B, U=A, and $v_{s}=0$. It is observed that none of links (0,j) for j=1,2,⋯,n can be members of *K*. Therefore, we may assume that only links (0,n+j) for j=1,2,⋯,n are removed. For convenience, link (0,n+j) is simply denoted by index *j*, and let *K* be any set of removed links that does not violate the budget constraint $\sum_{e\in K}w_{e}\leq R$. Then, $\sum_{j\in K}b_{j}\leq B$ and the total length of the shortest-path tree from vertex 0 becomes $\sum_{j\in K}a_{j}$. That is, there is a one to one correspondence between solutions of LG-KRP and solutions of KNAP. □

Let $V_{s}\left(t\right)$ be the set of infected vertexes at stage *t*, and $V_{d}\left(t\right)$ denote the newly detected set of infected vertexes at *t*. The MPC strategy for DSPTI problem is shown in Algorithm 2, named as MPC-DSPTI strategy.
**Algorithm 2** MPC-DSPTI: MPC Strategy for DSPTI1:Initialize $\hat{Y}\leftarrow \emptyset$, $\underline{z}\leftarrow -\infty$, $\bar{z}\leftarrow +\infty$, $\hat{x}\leftarrow 0$, t←02:**while**$|V_{s}\left|<|V\right|$**do**3:      Malware Detection: obtain a set $V_{d}\left(t\right)$4:      **if** $V_{d}\left(t\right)\neq \emptyset$ **then**5:         Defender’s Decision: solve LG-SSPTI(*t*) for $x^{t}$6:         Malware Interdiction: allocate countermeasures         according to $x^{t}$7:      Attacker’s Decision: solve [Sub($x^{t}$)] for $y^{t}$8:      Malware propagation: penetrate the CPS based on $y^{t}$9:      t←t+1


Although LG-SSPTI problem is still NP-hard, two benefits are brought by introducing the local-greedy allocation constraint. (1) The number of decision variables x is reduced a lot and will not increase directly as the growth of graph *G*, thereby expanding the solvable scale of the problem. (2) Since only urgent interdiction demands at current stage (i.e., the needs to allocate countermeasures to links that may be penetrated immediately by the attacker) are satisfied, and most resources remain available for future interdiction actions. That is, using this MPC strategy the defender can adopt an observe-and-response decision adaptively. This helps the defender reduce the decision-making risk due to the uncertainty of the distribution of malwares which is essential for avoiding countermeasures resources waste and achieving more robust decisions.

Due to the similarity between LG-SSPTI and SSPTI, the proposed algorithm S-BD can be used to solve LG-SSPTI optimally by adding the Constraint (17) to the the [Master($\hat{Y}$)] in Section 4. In this MPC strategy, the solution of any instance of LG-SSPTI can be viewed as a kind of approximate solution of the SSPTI with the same constraints except the Constraint (17). Let $\delta =\max_{e\in E}\frac{d_{e}}{c_{e}}$, and the approximate performance analysis is shown in Theorem 5, which proves that LG-SSPTI holds an 1+δ approximation for SSPTI.

**Theorem** **5.** *The solution of LG-SSPTI can achieve the 1+δ approximation for the corresponding SSPTI problem.*

**Proof** **of** **Theorem** **5.** Without loss of generality, we omit the superscript “*t*” in [LG-SSPTI(*t*)] during this proof. Let $X_{L}$ and $Y_{L}$ be the feasible region of x and y determined by Constraints (14)–(17) in LG-SSPTI, respectively. The optimal solution of SSPTI is $u^{*}=\max_{x\in X}\min_{y\in Y}U\left(x,y\right)=U\left(x^{*},y^{*}\right)$, and let $u_{L}^{*}=\max_{x\in X_{L}}\min_{y\in Y_{L}}U\left(x,y\right)=U\left(x_{L}^{*},y_{L}^{*}\right)$ be the optimal solution of LG-SSPTI.

Firstly, it is clear that $Y_{L}=Y$, and $X_{L}\subseteq X$; thus,

(18) $$u^{*}=\max_{x\in X}\min_{y\in Y}\;U\left(x,y\right)\geq \max_{x\in X_{L}}\min_{y\in Y}\;U\left(x,y\right)=u_{L}^{*}.$$

Then, we define $u_{L}^{\prime }=U\left(x^{*},y_{L}^{*}\right)$. For the fixed $x^{*}$, $u^{*}=\min_{y\in Y}U\left(x^{*},y\right)$; hence, for the specific $y_{L}^{*}\in Y$, we have

(19) $$u_{L}^{\prime }\geq u^{*}.$$

Let $x^{*}=x^{a*}+x^{b*}$, where $x_{e}^{a*}=x_{e}^{*},\;\forall \;e\in NS\left(v_{s}\right)$, else $x_{e}^{a*}=0$. For the fixed $y_{L}^{*}$, we have the explicit expression of $u_{L}^{\prime }$ as follows:

(20) $$\begin{array}{ccc} u_{L}^{*} & = & c^{T}Hy_{L}^{*}+x_{L}^{*T}DHy_{L}^{*}, \end{array}$$

(21) $$\begin{array}{ccc} u_{L}^{\prime } & = & c^{T}Hy_{L}^{*}+x^{*T}DHy_{L}^{*}, \end{array}$$

(22) $$\begin{array}{ccc} & = & c^{T}Hy_{L}^{*}+x^{a*T}DHy_{L}^{*}+x^{b*T}DHy_{L}^{*}. \end{array}$$

Since $x^{a*T}\in X_{L}$, $y_{L}^{*}\in Y$, we have $U\left(x^{a*},y_{L}^{*}\right)\leq U\left(x_{L}^{*},y_{L}^{*}\right)$, i.e.,

(23) $$c^{T}Hy_{L}^{*}+x^{a*T}DHy_{L}^{*}\leq u_{L}^{*}.$$

On the other hand, item $x^{b*T}DHy_{L}^{*}$ in Equation (22) is the sum of added path lengths on tree $y_{L}^{*}$ except the added path length of links $e\in NS(v_{s})$. Since each link can only be interdicted once at most, by assuming that all the links on tree $y_{L}^{*}$ could be interdicted by the defender, we could obtain a upper bound:

(24) $$x^{b*T}DHy_{L}^{*}<d^{T}Hy_{L}^{*}\leq \delta \cdot c^{T}Hy_{L}^{*}.$$

Meanwhile, it is clear that

(25) $$\delta \cdot c^{T}Hy_{L}^{*}\leq \delta \cdot \left(c^{T}Hy_{L}^{*}+x_{L}^{*T}DHy_{L}^{*}\right)=\delta \cdot u_{L}^{*}.$$

Finally, we conclude that $\left(1+\delta \right)u_{L}^{*}>u_{L}^{\prime }\geq u^{*}$, i.e.,

(26) $$\frac{u^{*}}{u_{L}^{*}}<1+\delta .$$

□

## 6. Performance Evaluation

This section first introduces several defense strategies, including existing strategies and the proposed strategy. Then, we introduce the performance metrics for both static and dynamic strategies, and the evaluation settings in the experiment are given including simulation methods of networks and information of the real-case CPS network. After that, we evaluate our proposed algorithms and strategies through extensive experiments with static and dynamic settings.

### 6.1. Defense Strategies

We first evaluate two static defense strategies, i.e., SSPTI and LG-SSPTI. The defender who adopts static strategies allocates all available defense resources in an once-and-for-all manner, once infected devices are detected. Then, three different dynamic strategies are considered as follows for evaluation with respect to the pay-off between CPS security and safety.

1) We propose the static shortest-path tree interdiction (SSPTI) game, model it as a bi-level integer p A fail-safe strategy (FSA): when a device is infected and detected during the unprotected exposure, it will be isolated from the CPS. Although its neighbors may have been infected, as well, no light-weight countermeasures will be taken on them such that fail-safe ability can be maintained as much as possible.
2) A fail-secure strategy (FSE): when a device is infected and detected during the unprotected exposure, the device itself and its neighbors will be isolated at the same time so as to avoid further infections deriving from possible infected neighbors. Hence, fail-secure ability is the first priority for the defender.
3) The MPC strategy (MPC): as mentioned in Section 5, when a device is infected and detected during the unprotected exposure, it will be isolated and then light-weight countermeasures will be allocated optimally to its neighbors by solving a LG-SSPTI problem. In fact, this strategy intends to achieve a balance between fail-safe ability and fail-secure ability in CPS defense.

### 6.2. Performance Metrics and Evaluation Settings

We first use the following metrics for performance comparison in the evaluation of static strategies on different types of networks:

1) The achieved objective *u* under different static strategies. That is, the shortest-path three length which the attacker can achieved under the situation of defenders’ countermeasure implementation. The larger *u* the attacker gains, the more effectively the defender defends in the malware propagation. We mainly compare the achieved *u* of SSPTI and LG-SSPTI, and analyze the impact of δ on the actual approximate ratio.
2) The algorithm running time. We compare the running time of SSPTI and LG-SSPTI by changing the scale of the problem.

In the experiments, we select three representative types of CPS networks for evaluation, i.e., square-lattice networks, standard Erd$\ddot{o}$s-Rényi (ER) networks [51] and scale-free networks [52]. Lattice networks, where each vertex has the same number of links, have been used to model physical systems [2], such as water supply systems, power grid systems, etc. ER network is the original model of networks based on random graph theory, which has a Poisson degree distribution. Many more real-life networks obey a power-law form in degree distribution, such as the Internet [53], mobile communication networks [54], and airline networks [55]. These systems can be approximately modeled as scale-free networks.

For evaluation, 100 instances of square-lattice networks, ER networks and scale-free networks with 50 vertexes, 85, 178 (in average), and 144 links are generated, respectively, in the evaluation of static strategies. An initial infectious device is randomly generated to evaluate the performance of static strategies. In each instance, link attributes c, w are randomly generated and uniformly distributed on [1,5]. Node attributes r is uniformly distributed on [1,10]. Let interdiction delay d=δ·c so as to analyse the impact of δ. Then, a set of square-lattice networks, ER networks and scale-free networks of different size (from 50 vertexes to 1600 vertexes) are generated with the same link attributes setting to compare the running time. Here, the upper bound of total loss *R* is set as $0.5\cdot \sum_{e\in E}w_{e}$, and δ is set as 0.6 to consider the impact of network size on static strategies.

Additionally, the followings metrics are used to evaluate the performance of different dynamic defense strategies, which cares for the gain and loss of deploying light-weight countermeasures against stealth malware propagation in CPS.

1) The speed and scale of malware propagation, which represents the security situation of the CPS during the defense process.
2) The size of giant component of the CPS during the defense process, which is a major indicator of safety situation for networked systems.

According to the features of stealth malware detection [12], we can simply assume that the probability that an infected vertex can be detected by the defender grows linearly as the increase of its infection duration, and after a given duration $T_{d}$ it can be certainly detected. The initial ratio of infection is set to be 2% in the experiments that obtain the metrics versus time curves, and then varies from 1% to 100% in order to test the difference of steady infection state (i.e., the final scope of infection) under different initial infection ratios. Here, 100 instances of square-lattice networks, ER networks and scale-free networks with 300 vertexes are generated, respectively, with the same link attributes setting (e.g., c, w, r), and let δ=1. We set the upper bound of total loss $R=0.5\cdot \sum_{e\in E}w_{e}$, and the individual upper bound of loss $r_{i}=0.8\cdot \sum_{e\in NS(i)}w_{e}$ so as to meet the safety requirement of CPS. Moreover, a practical CPS case is used to evaluate the dynamic strategies, i.e., Italian coupled communication and power grid network [56]. There are 39 vertexes and 102 links in the control and communication network, 310 vertexes and 361 links in the power grid network, and 169 coupled links between them. We execute the evaluation 100 times adopting the above link attributes setting and average the numerical results.

We program our algorithms using YALMIP toolbox [57] and Gurobi version 7.0.2 callable library for exact solution for master problems. Computation is performed on a Windows10 (64) computer with 2.40 GHz Intel(R) Core i5 CPU and 4.0G RAM.

### 6.3. Evaluation Results of Static Strategies

#### 6.3.1. Comparison of Achieved Objective *u*

The performance comparison of static strategies SSPTI and LG-SSPTI on 100 instances of three types of networks with 50 vertexes is shown in Table 2. It is obvious that as the increase of δ from 0.2 to 1.0 the average actual approximation ratio $\bar{\frac{u^{*}}{u_{L}^{*}}}$ on square-lattice networks rises from 1.08 to 1.33, whereas that on ER networks and scale-free networks grows slightly from 1.03 to 1.13 and 1.11, respectively. That is, LG-SSPTI strategy can actually achieve a better approximation ratio, though the approximation ratio given by Theorem 5 is 1+δ, especially on scale-free networks. The length of shortest-path tree for malware propagation on square-lattice networks is noticeably larger than that on ER and scale-free networks with the same nodes scale. Besides, the consumed resources $R_{u}$ when adopting SSPTI strategy is nearly 3 times of that when adopting LG-SSPTI strategy on ER and scale-free networks, and around 7 times of that on square-lattice networks. The result demonstrates that LG-SSPTI strategy can save interdiction resources in a large amount compared with SSPTI, and these saved resources can be used in the future shots of defense thereby bringing extra payoffs.

In summary, the defender who adopts LG-SSPTI strategy can achieve at most 1+δ approximation of SSPTI strategy and consume quite fewer resources compared with SSPTI strategy in the meantime on square-lattice networks, ER networks and scale-free networks. Additionally, a malware can be spread faster in the manner of shortest-path tree on ER and scale-free networks compared with square-lattice networks, deriving from the structure difference between them.

#### 6.3.2. Comparison of Algorithm Running Time

In Table 3, we compare the algorithm running time of SSPTI and LG-SSPTI on three types of networks. For each number of vertexes |V|, 10 instances are generated for evaluation. With the enlargement of the scale of a problem especially the number of links |E|, the average running time of both strategies increases by various extents. When the scale of networks increase to over 200 links, SSPTI cannot solve all instances optimally in the given time (1800 CPU seconds) on three kinds of networks. However, compared with SSPTI, the solvable scale of LG-SSPTI has increased to 6268, 1479, and 4794 links on square-lattice, ER, and scale-free networks, respectively, and LG-SSPTI is faster than SSPTI by a wide margin. The superiority of LG-SSPTI is rooted in the fact that its decision variables will not increase directly as the growth of network. Since the number of an infected node’s neighbors on square-lattice network is a constant except that of edge nodes, the running time increases slowly as the rise of network size, and the average number of iterations in the decomposition procedure keeps nearly the same. The degree distribution of scale-free networks follows a power low asymptotically, and randomly selected initial infectious nodes usually have a low degree in statistical; hence, the LG-SSPTI running time on scale-free networks is lower than its counterpart on ER networks, which approximately holds Poisson degree distributions.

Therefore, we conclude that LG-SSPTI expands the solvable scale of the problem to a large extent on three kinds of generated networks. Besides, the running time of LG-SSPTI on square-lattice networks and scale-free networks is lower than it counterpart on ER networks.

### 6.4. Evaluation Results of Dynamic Strategies

#### 6.4.1. Performance on Generated Networks

**Figure 4:**
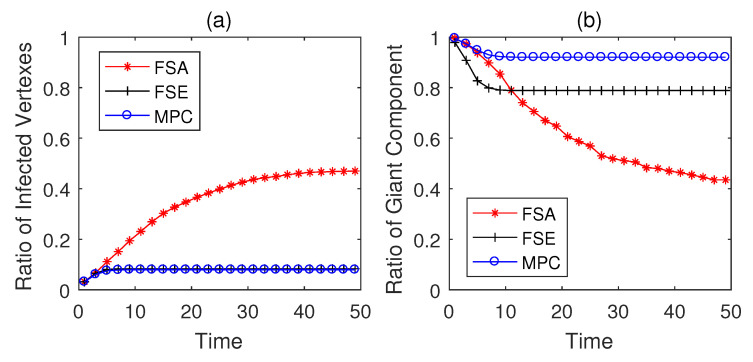
Performance of dynamic defense strategies on square-lattice networks (|E|=563, $T_{d}=5$). (**a**) Security situation; (**b**) safety situation.

**Figure 5:**
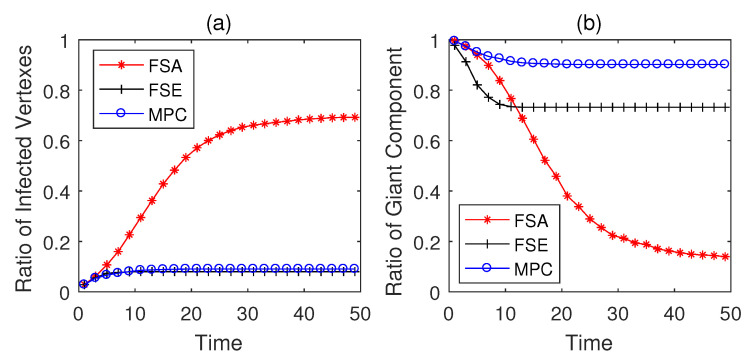
Performance of dynamic defense strategies on Erd$\ddot{o}$s-Rényi (ER) networks ($|\bar{E}|=526$, $T_{d}=5$). (**a**) Security situation; (**b**) safety situation.

**Figure 6:**
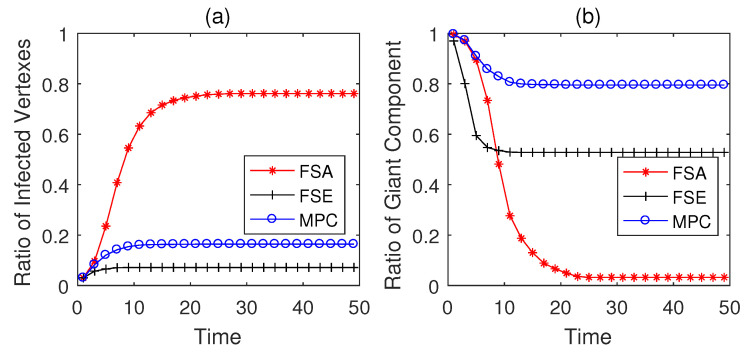
Performance of dynamic defense strategies on scale-free networks (|E|=597, $T_{d}=5$). (**a**) Security situation; (**b**) safety situation.

The propagation performances of three dynamic defense strategies on square-lattice networks, ER networks and scale-free networks are shown in Figure 4, Figure 5 and Figure 6. From the security situation results in subplot (a), our proposed strategy MPC and the fail-secure strategy FSE can effectively retard and mitigate the propagation of malwares and all the ratios of infected vertexes are suppressed fewer than 20%, while the fail-safe strategy FSA cannot prevent malware penetration effectively resulting a higher propagation speed and a larger scope of infection. Meanwhile, scale-free networks tend to be more fragile towards malware propagation no matter which defense strategies the defender adopts. From the safety situation results in subplot (b), the proposed MPC strategy slows the collapse of giant component ratio $r_{gc}$ down and maintains the highest ratio of giant component among three dynamic strategies. At the beginning of the infection, FSA holds a lower collapse rate of $r_{gc}$ compared with that of FSE; however, due to the challenge of stealth malware quick detection, the propagation cannot be controlled in the short term resulting the outbreak of newly infected vertexes in the medium term. Hence, safety cannot be guaranteed as expected without active actions on system security, due to the interdependence between safety and security in CPS. The FSE strategy, on the other hand, can control the propagation of malware, while it gives rise to a sharp drop in $r_{gc}$ at early stages (lower than 60% in Figure 6b). Althougfh, FSE outperforms in maintaining the security situation of CPS before the implementation of heavy-weight countermeasures, the high collapse rate of $r_{gc}$ of FSE at the beginning of infection is likely to make a large loss on safety assurance.

Therefore, among these three strategies, our proposed MPC strategy can align CPS security and safety properly. Although the FSA strategy aims to maintain safety and reduce the loss on system functionalities as much as possible, more vertexes will be infected in the future due to the undetected threats and the unprotected exposure, resulting worse performance on both safety and security maintenance. FSE, on the other hand, can achieve a low infected ratio but fail to maintain the connectivity and safety of CPS.

#### 6.4.2. Performance on Real CPS Cases

**Figure 7:**
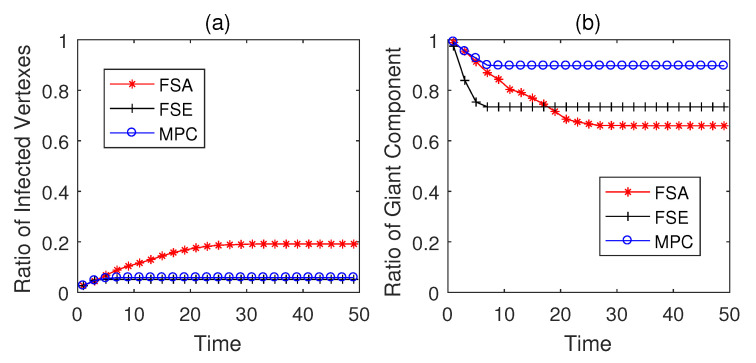
Performance of dynamic defense strategies on Italian coupled communication and power grid network ($T_{d}=5$). (**a**) Security situation; (**b**) safety situation.

**Figure 8:**
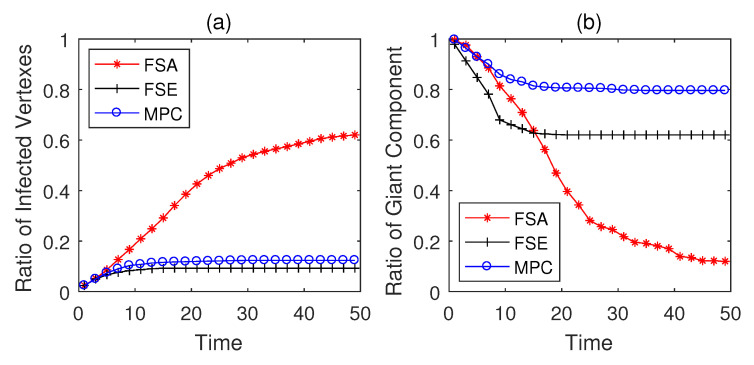
Performance of dynamic defense strategies on Italian coupled communication and power grid network ($T_{d}=10$). (**a**) Security situation; (**b**) safety situation.

**Figure 9:**
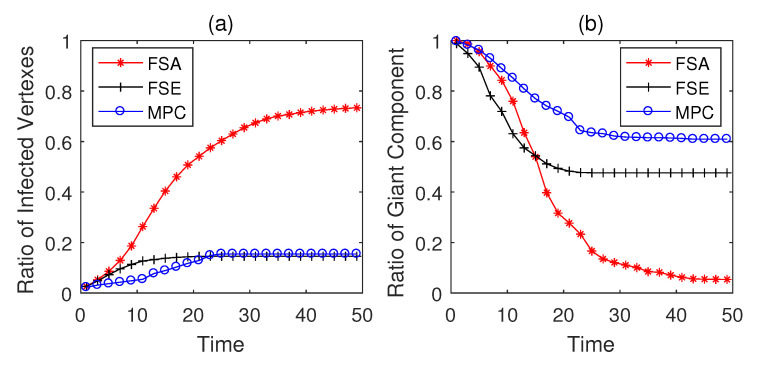
Performance of dynamic defense strategies on Italian coupled communication and power grid network ($T_{d}=15$). (**a**) Security situation; (**b**) safety situation.

The simulation results on Italian coupled communication and power grid network are shown in Figure 7, Figure 8 and Figure 9. From the results in Figure 7, the performances of three strategies are similar to the above conclusions on generated networks, and MPC strategy performs better than the other two strategies in both security and safety assurance. When $T_{d}=5$, the practical CPS network tends to be less fragile than the generated networks due to the difference in network structures. However, as the increase of $T_{d}$, i.e., the rise of detection difficulty, the performance of all strategies degenerates (see in Figure 8 and Figure 9). Both MPC and FSE strategy have a slight rise in the ratio of infected vertexes, while the spread of malwares cannot be effectively controlled by FSA strategy. The $r_{gc}$ when adopting MPC strategy decreases at the lowest speed and can be controlled in a relatively higher level among three strategies.

As a result, the performance of all strategies degenerates as the increase of detection difficulty. However, the proposed MPC strategy still outperforms other strategies, and it can help retard the spread of malwares and the cascade of devices failure during the time-span of unprotected exposure, thereby achieving a balance between security and safety.

#### 6.4.3. Discussion of Steady Infection State

**Figure 10:**
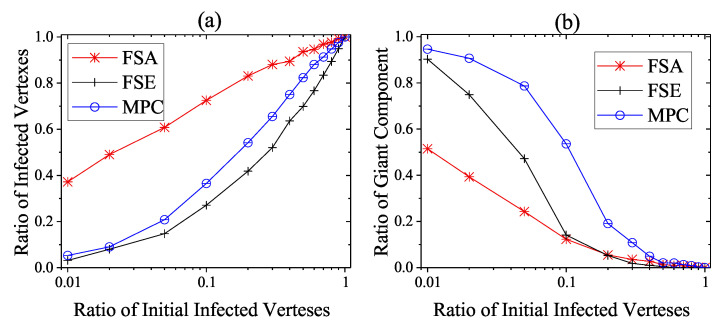
Steady State of dynamic defense on square-lattice networks against various initial infected vertexes ratios. (**a**) Security situation; (**b**) safety situation.

**Figure 11:**
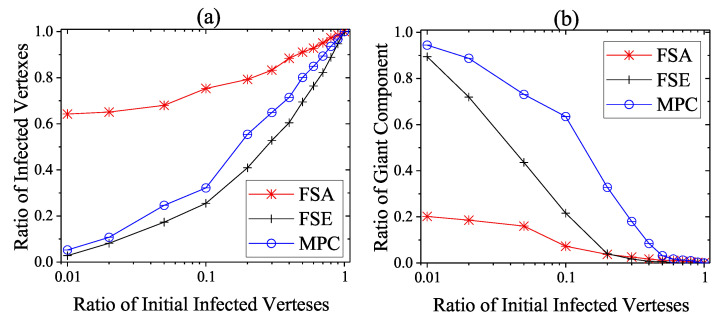
Steady state of dynamic defense on ER networks against various initial infected vertexes ratios. (**a**) Security situation; (**b**) safety situation.

**Figure 12:**
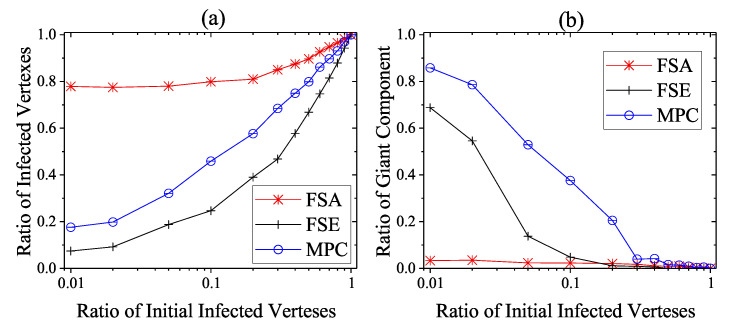
Steady state of dynamic defense on scale-free networks against various initial infected vertexes ratios. (**a**) Security situation; (**b**) safety situation.

We then consider the extreme case that no heavy-weight countermeasures are developed and deployed until the steady infection state, i.e., the unprotected exposure is relatively large and only light-weight countermeasures work. This situation is not very common, but considering the cunning techniques of attackers in reality CPS defenders need to take actions in case of emergency. As shown in Figure 10, Figure 11 and Figure 12, the performance of dynamic strategies on the final scope of infection with various initial infection ratios are considered on three types of networks. Here, the detection time threshold $T_{d}$ is set to be 5. As the increase of initial infection ratio, the average steady scope of infection grows to 100% with different rate and the average steady $r_{gc}$ falls to 0%. More specifically, it is clear that FSE and MPC outperform FSA on the steady state of both security and safety situation among three kinds of networks. When the initial infection ratio is 1%, the average final ratio of infected vertexes of FSA is around 38%, 64%, and 78% on square-lattice networks, ER networks and scale-free networks, respectively, while that of MPC and FSE is below 10% and 20%. The performance on $r_{gc}$ assurance of FSA is much worse than MPC and FSE, reporting around 51%, 20%, and 3%, respectively, when the initial infection ratio is 1%, whereas that of MPC and FSE is more than 90% and 70%.

The performance of FSE is slightly better than that of MPC in reducing the scope of infection, but FSE is intrinsically inferior to MPC in safety assurance of CPS. The superiority of FSE in final ratio of infection over MPC is not more than 15% on square-lattice networks and ER networks and 25% on scare-free networks, but MPC can sometimes exceed FES around 45% in the steady $r_{gc}$. More significantly, even though more vertexes are immune to malwares when using FSE rather than MPC, the CPS is not likely to function well at all because of the extremely fragmented condition. For instance, in Figure 12 when initial infected ratio is 10%, FSE outperforms MPC in final ratio of infection reporting nearly 25% versus 46%; however, the corresponding $r_{gc}$ of FSE and MPC is about 5% and 38%. That is, the CPS adopting FSE becomes a combination of isolated vertexes and network fragments which cannot supports most of the function requirements, although around 75% of these components are not infected. On the other hand, the CPS adopting MPC still has 54% of vertexes which are not infected; meanwhile, 38% vertexes are in the same giant component and belong to the set of 54% uninfected vertexes, i.e., this giant component is protected to be secure and safety at the same time.

**Figure 13:**
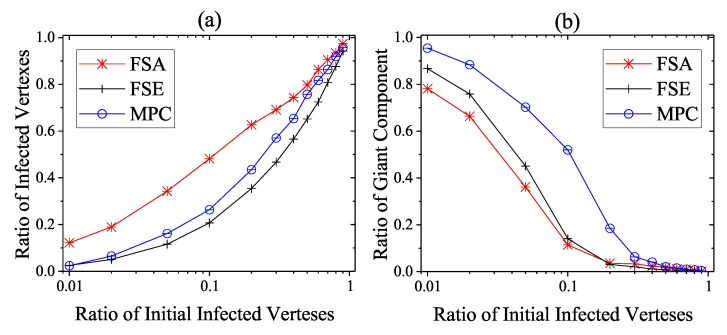
Steady state of dynamic defense on Italian coupled communication and power grid network against various initial infected vertexes ratios. (**a**) Security situation; (**b**) safety situation.

Similar results on Italian coupled communication and power grid network can be obtained when the extreme case is taking account, shown in Figure 13. MPC is still the best choice when considering the balance between fail-secure ability and fail-safe ability among those three dynamic defense strategies. The performance of FSE is slightly better than that of MPC in reducing the scope of infection where the gap between is not more than 10%. MPC is intrinsically superior to FSE in safety assurance of CPS, reporting 52% versus 14% in final $r_{gc}$. Besides, the inferiority of FSA to the other strategies on Italian coupled network is not very large compared to that on generated networks. However, based on the results in Figure 7, Figure 8 and Figure 9, we can conclude that the performance difference between different strategies will become more distinctive as the increase of detection difficulty.

To summarize, considering the extreme case that the unprotected exposure is relatively large and only light-weight countermeasures work, MPC is the best choice when both fail-secure and fail-safe ability is essential among those dynamic strategies on both generated and real-case CPS networks. The reason lies on the fact that a defender who adopts MPC can reduce the scope of final infection and maintain the communication and other normal functions of the giant component to a large extent. Therefore, when the defender faces a stealth APT attack with fewer heavy-weight countermeasures to defense the CPS in an emergency, the proposed MPC strategy can provide a option of using light-weight countermeasures so as to achieve a balance between fail-secure ability and fail-safe ability while retarding the stealth malware propagation in CPS.

## 7. Conclusions

In this work, we focus on the static and dynamic defense against stealth malware propagation in Cyber-Physical Systems. We first modeled the attack-and-defense process between the CPS defender and attacker as a static shortest-path tree interdiction game, and formulated it as a bi-level linear integer programming. To meet the real-time decision-making requirement, we extended SSPTI game to an extensive-form game, i.e., DSPTI. The NP-hardness of SSPTI and DSPTI was systematically analyzed, and optimal algorithms were designed, as well. In order to find an optimal trade-off between the gain and loss of deploying light-weight countermeasures, we proposed a dynamic defense strategy, i.e., MPC strategy. Extensive experiments have been conducted on both simulated and real-case based CPS networks, which demonstrates the efficiency of the proposed algorithms. Furthermore, those evaluation results illustrated that the defender can achieve a balance between fail-secure ability and fail-safe ability while retarding the stealth malware propagation in CPS. Although, the application of this framework in the practice of CPS defense still needs a lot meticulous research and work, we believe that it will provide valuable enlightenment for subsequent research in the future.

## Figures and Tables

**Table 1:** Notations of main parameters and variables.

Notations		Descriptions
Sets	$N_{p}$	The set of players, i.e., a defender and an attacker
	*V*	The set of devices (vertexes) in the CPS network *G*. $v_{i}\in V$ where i={1,2,⋯,|V|}
	*E*	The set of links in *G*. Each link e=(i,j)∈E, $v_{i},v_{j}\in V$
Parameters	$c_{e}$	Cost for an attacker to propagate a malware through the link *e*. $c_{e}\geq 0$ (vector form c)
	$d_{e}$	Delay on link *e* if it is interdicted by the defender (vector form d)
	$w_{e}$	Safety loss of link *e* if it is interdicted by the defender (vector form w)
	*R*	Upper bound of total safety loss for the safe of the whole CPS
	$r_{i}$	Individual upper bound of safety loss for the vertex *i*
	*u*	The utility function for game players
Decision variables	$x_{e}$	The defender’s decision variable (vector form x), where $x_{e}=1$ if the link *e* is interdicted; otherwise $x_{e}=0$
	$y_{e}$	The attacker’s decision variable (vector form y) $y_{e}=1$ if the link *e* is chosen to pass through; otherwise $y_{e}=0$

**Table 2:** Comparison of static strategies performance.

Network Type	δ	SSPTI			LG-SSPTI			$\bar{\frac{u^{*}}{u_{L}^{*}}}$
		${\bar{u}}^{*}$	σ	$R_{u}$	${\bar{u}}^{*}$	σ	$R_{u}$	
Square-lattice Networks	0.2	520.17	151.08	64.70	479.60	137.83	8.45	1.08
	0.4	561.65	102.14	66.25	496.84	83.61	9.40	1.13
	0.6	630.09	158.66	72.45	517.23	123.83	8.75	1.21
	0.8	654.76	125.12	73.80	513.53	97.92	10.15	1.28
	1.0	677.50	161.87	80.50	505.80	107.72	10.90	1.33
Erd$\ddot{o}$s-Rényi Networks	0.2	156.72	37.23	56.25	152.04	35.45	24.45	1.03
	0.4	183.47	33.08	62.05	171.98	30.05	19.40	1.07
	0.6	190.27	35.14	72.15	172.89	29.71	23.75	1.10
	0.8	193.12	37.56	70.65	173.45	30.60	22.40	1.11
	1.0	201.55	32.77	74.40	178.20	28.59	24.10	1.13
Scale-free Networks	0.2	151.53	33.50	51.25	146.74	31.61	17.35	1.03
	0.4	161.00	37.63	50.50	151.65	33.69	18.90	1.06
	0.6	165.66	43.22	60.05	153.19	37.19	21.55	1.08
	0.8	202.80	47.90	59.00	181.40	40.31	15.90	1.11
	1.0	191.80	33.92	61.05	173.10	29.01	19.95	1.11

Superscript “ $\bar{}$ ” denotes the average of related parameters; σ denotes the standard deviation of $u^{*}$ and $u_{L}^{*}$; $R_{u}$ denotes the consumed resources; *R* is set to be 200.

**Table 3:** Comparison of running time (static shortest-path tree interdiction (SSPTI) vs. local-greedy (LG)-SSPTI).

Network Type	Problem		SSPTI			LG-SSPTI		
	|V|	|E|	T	σ	I	T	σ	I
Square-lattice Networks	50	85	15.64	8.43	12	2.80	0.84	3
	100	171	36.61	15.12	21	5.19	2.14	4
	200	367	[8]	–	–	6.33	1.93	3
	400	742	[0]	–	–	13.52	5.97	3
	800	1534	[0]	–	–	27.08	10.41	3
	1600	3084	[0]	–	–	55.74	20.84	3
	3200	6268	[0]	–	–	235.25	47.77	3
Erd$\ddot{o}$s-Rényi Networks	50	178	36.43	20.25	30	4.68	1.61	6
	100	368	[4]	–	–	27.68	20.68	15
	200	744	[0]	–	–	89.90	59.39	33
	400	1479	[0]	–	–	592.19	731.97	64
	800	2958	[0]	–	–	[8]	–	–
	1600	5944	[0]	–	–	[5]	–	–
	3200	11922	[0]	–	–	[0]	–	–
Scale-free Networks	50	144	202.82	430.48	56	12.54	10.73	12
	100	294	[1]	–	–	25.04	33.98	14
	200	594	[0]	–	–	26.16	12.42	10
	400	1194	[0]	–	–	69.48	111.19	14
	800	2394	[0]	–	–	308.63	561.78	20
	1600	4794	[0]	–	–	483.07	341.00	12
	3200	9594	[0]	–	–	[8]	–	–

Nos. in the square brackets presents the number of solvable problems under 1800 CPU second from 10 instances. “–” indicates “not applicable”, i.e., at least one of the 10 instances cannot be solved optimally in the allotted time. *T*, average running time for 10 instances, in CPU seconds; σ, standard deviation of *T* in CPU seconds; *I*, average number of iterations for decomposition algorithms.
